# Pan-Genome-Scale Metabolic Reconstruction Reveals Conserved Metabolic Functions in *Candida albicans*

**DOI:** 10.3390/jof12090697

**Published:** 2026-09-17

**Authors:** Ya Meng, Yiming Zhang, Lei Zhang

**Affiliations:** 1 Microbiome-X, School of Public Health, Cheeloo College of Medicine, Shandong University, Jinan 250012, China; mengya@mail.sdu.edu.cn (Y.M.); z1406056750@163.com (Y.Z.); 2 School of Pharmaceutical Engineering, Jining Medical University, Jining 272002, China

**Keywords:** *Candida albicans*, pan-genome, pan-GEM, strain-specific GEM, gene essentiality, vaginal-like synthetic medium

## Abstract

*Candida albicans* is a major cause of human mucosal and invasive fungal infections, but the relationship between its intraspecific genomic diversity and metabolic variation remains poorly understood. Here, we integrated 80 public *C. albicans* genome assemblies, published fungal genome-scale metabolic models (GEMs), public reaction databases, and orthogroup-linked gene–protein–reaction (GPR) evidence to construct a species-level *C. albicans* pan-GEM and derived 80 strain-specific GEMs (ssGEMs) through genome projection. The pan-genome comprised 10,308 orthogroups, including 4215 core, 5947 accessory, and 146 singleton orthogroups. The final pan-GEM contained 1986 reactions, 1777 metabolites, and 865 genes. After feasibility rescue, all 80 ssGEMs met the feasibility criterion for predicted growth and passed the closed-uptake energy-generating-cycle test. Among experimentally essential genes with resolvable GPR associations, 23 were consistently predicted as model-essential across all final ssGEMs. As an application of the ssGEM collection, nutrient-boundary simulations showed that increasing D-glucose uptake markedly increased predicted growth across 79 feasible ssGEMs. This framework provides a reusable resource for comparing conserved metabolic functions and genome-projected reaction differences across *C. albicans* strains.

## 1. Introduction

Invasive and chronic fungal infections impose a persistent burden on clinical care and public health. *Candida* species are among the most common groups of human fungal pathogens; *C. albicans* in particular can persist as a long-term commensal of the oral cavity, gut, and female reproductive tract, while also causing mucosal and invasive infection when host immune status, local microbiota, or nutrient availability shift [1,2,3,4]. This transition from colonization to pathogenicity is not determined by host factors alone; strain-level genetic differences may also shape environmental adaptation and drug response.

Prior studies have shown that *C. albicans* exhibits substantial intraspecies diversity in chromosomal structure, copy number, heterozygosity, recombination, and gene content [5,6,7]. These studies have provided a foundation for understanding the population structure and evolutionary history of *C. albicans*, but most analyses remain at the level of variant sites, gene families, or phylogeny, leaving a systematic investigation of *C. albicans* functional metabolism comparatively underexplored.

Genome-scale metabolic models (GEMs) mathematically organize metabolites, reactions, and gene–protein–reaction (GPR) associations into computable networks, thereby translating genomic information into testable metabolic hypotheses. Published *C. albicans* models, including iRV781 and subsequent host-associated reconstructions, have been applied to carbon/nitrogen source utilization, gene essentiality, host colonization, and fungal–bacterial interaction studies [8,9,10]. However, these models represent individual reference backgrounds with fixed gene and reaction repertoires, which cannot distinguish species-wide conserved reactions from reactions supported only in a subset of strains, or propagate gene-family presence and absence into comparable strain-specific networks.

Pan-genome metabolic modeling offers a suitable framework for addressing this gap. The *Gardnerella* pan-GEM integrated strain-level gene and reaction conservation, computational gene essentiality, and flux comparisons under vaginal nutrient conditions within a single model system [11]. The *Lactobacillaceae* PanGEM further demonstrated how comparable metabolic models can be constructed across a large genome collection [12]. Pan-genome studies of model fungi indicate that *C. albicans* also retains a substantial non-core gene complement, which may be linked to adaptation, pathogenicity-associated traits, and antimicrobial tolerance [13]. In fungal metabolic modeling, the *A. fumigatus* pan-GEM work has shown that species-level models can serve as reaction templates and be further decomposed into strain-specific models for comparative analysis [14]. Despite these advances, a curated *C. albicans* pan-GEM linked to a systematically reconstructed panel of strain-specific GEMs has been lacking.

**Figure 1:**
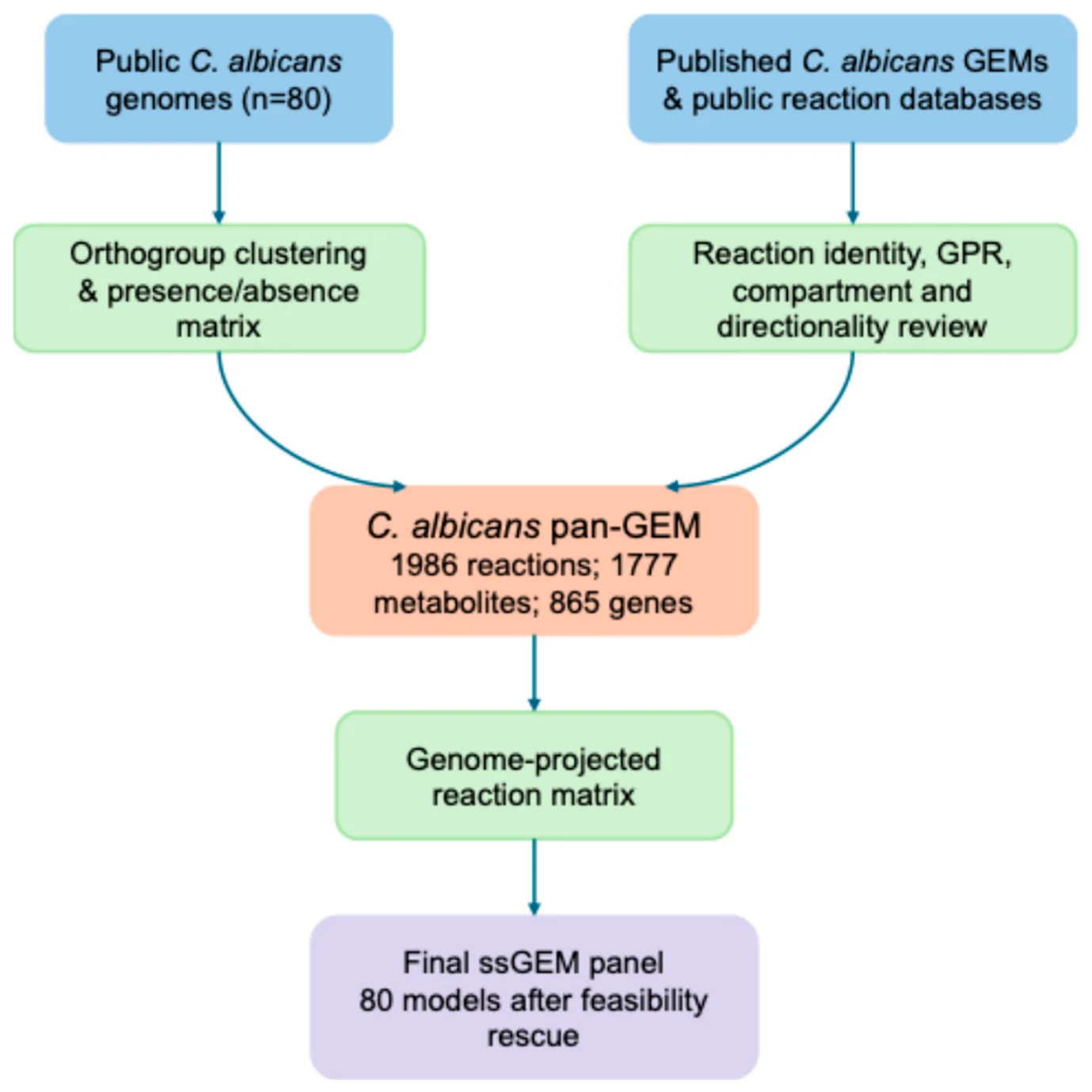
Workflow for construction of the *Candida albicans* pan-GEM and ssGEM collection.

In this study, we integrated 80 public *C. albicans* genome assemblies with published *C. albicans* and other fungal GEMs, public reaction databases, and orthogroup-linked GPR evidence to construct a *C. albicans* pan-GEM and derive 80 ssGEMs through genome projection, with feasibility rescue required for 72 of the initial drafts. We then used a vaginal-like synthetic medium to analyze computational gene essentiality and nutrient-boundary responses, evaluating the utility of this model resource for comparing conserved metabolic functions and genome-projected reaction differences. The overall reconstruction and analysis workflow is summarized in Figure 1.

## 2. Materials and Methods

### 2.1. Public Genome Collection, Gene Prediction, and Pan-Genome Analysis

Publicly available *C. albicans* genome assemblies released on or before 31 December 2025 were collected from NCBI Assembly [15]. Assembly quality was evaluated using BUSCO v6.0.0 with the saccharomycetes_odb12 lineage dataset to assess completeness and the proportions of missing and duplicated BUSCOs, together with genome size, GC content, contig number, and N50 [16]. Assemblies were required to have BUSCO completeness of at least 93%, BUSCO missingness of no more than 6%, BUSCO duplication below 10%, a genome size of 13–17 Mb, a GC content of 31.0–34.5%, no more than 6000 contigs, and an N50 of at least 10,000 bp. The final dataset contained 80 *C. albicans* genome assemblies. BUSCO completeness ranged from 93.0% to 98.7%, BUSCO missingness from 1.0% to 5.0%, contig number from 8 to 4939, N50 from 14,761 to 2,451,893 bp, genome size from 13.43 to 15.73 Mb, and GC content from 31.45% to 33.84% (Table S1).

Metadata were collected from public records, including assembly accession, strain name, BioProject, BioSample, isolation source, sampling site, source type, clinical status, and country. Ambiguous or missing metadata fields were recorded as “unknown” rather than inferred.

Protein-coding genes were predicted for all 80 assemblies using funannotate v1.8.17 (https://github.com/nextgenusfs/funannotate (accessed on 13 September 2026), DOI: 10.5281/zenodo.1134477). *Candida albicans* was specified as the species, and saccharomycetes_odb10 was used as the BUSCO lineage. The resulting protein sequences were used for orthogroup inference. Orthogroups were constructed with OrthoFinder v3.1.2 in multiple-sequence-alignment mode [17]. MMseqs2 v18.8cc5c was used for protein sequence searches, and FastTree v2.2.0 was used for gene-tree inference [18,19]. Based on the orthogroup presence/absence matrix, orthogroups were classified as core (present in >95% of genomes, 77–80 genomes), accessory (present in 2–76 genomes), or singleton (present in only one genome). Pan-genome and core-genome accumulation curves were computed using 999 random permutations of assembly addition order. At each addition step, the cumulative pan-genome size, the number of orthogroups shared by all included assemblies, and the number of newly added orthogroups were recorded. Mean values and 95% empirical intervals, defined by the 2.5th and 97.5th percentiles, were calculated across permutations.

### 2.2. Functional Annotation and Candida-Associated Feature Screening

Functional annotations were assigned to the predicted protein sequences using eggNOG-mapper v2.1.13 with the eggNOG v5.0.2 database [20,21]. DIAMOND v2.1.24 was used by eggNOG-mapper with the options --itype proteins -m diamond --sensmode more-sensitive [18]. For each orthogroup, COG, EC, KEGG orthology, KEGG reaction, GO, PFAM, CAZy, and BiGG reaction information from member genes were aggregated. Enzyme/metabolic annotation and KEGG reaction annotation were used as the primary descriptors of functional distribution across the core, accessory, and singleton categories.

To identify functions relevant to *Candida* biology, orthogroups were screened using predefined keyword- and identifier-matching rules applied to member-protein descriptions and GO, KEGG, EC, and PFAM annotations. The screened categories included cell-wall adhesion, hyphal morphogenesis, drug efflux/resistance, oxidative stress, lipase/phospholipase activity, carbon utilization, sterol/ergosterol metabolism, and secreted protease. Antifungal-related orthogroups were further grouped into drug efflux transporters, azole target/sterol pathway, and echinocandin/cell-wall target genes. An orthogroup was assigned to a category when at least one annotation from its member proteins matched the corresponding rule, and multiple category assignments were permitted. The complete matching criteria are provided in Table S2. This screening provides descriptive annotation statistics and does not infer phenotypic traits.

### 2.3. Pan-GEM Construction and Reaction Evidence Integration

The pan-GEM was constructed using the refined *C. albicans* GEM reported by Hu et al. as the primary model scaffold [10]. The biomass equation was mainly based on iRV781 [8]. Mirhakkak2021_Calb_GSMM and the CarveFungi draft models served as auxiliary references for reaction identity, compartment assignment, transport reactions, and network connectivity [9,22]. The complete pan-GEM reaction, metabolite, and gene information, including the biomass formulation, is provided in Table S3.

KEGG, MetaCyc, BiGG Models, Rhea, and ChEBI were used to cross-check reaction identifiers, reaction definitions, substrates and products, stoichiometry, directionality, metabolite identity, chemical formulas, and charge [23,24,25,26,27]. Only candidate reactions with well-defined stoichiometry, clear biochemical rationale, and unambiguous reaction definitions were incorporated into the executable network; reactions supported only by database mapping, with conflicting sources, or with ambiguous metabolite definitions were retained as candidates for further manual curation.

Compartmentalization was curated by combining reference-model records, reaction biochemistry, and protein localization predictions. For proteins with ambiguous compartment assignment, DeepLoc 2.0 was used to predict subcellular localization, in conjunction with reaction type, metabolite location, and database annotation [28]. Biomass, exchange reactions, boundary conditions, and reaction directionality were manually curated to ensure the model could support FBA, pFBA, and gene-deletion analysis under unified conditions.

### 2.4. ssGEM Generation, Feasibility Rescue, and Quality Control

After mapping pan-GEM genes to orthogroups, GPR rules were evaluated for each assembly using the orthogroup presence/absence matrix. For an AND rule, all required genes had to be present, whereas an OR rule required at least one gene to be present. An initial genome-projected draft was generated for each assembly by retaining GPR-associated reactions whose rules were satisfied and removing those whose rules were not satisfied. Reactions without GPRs were retained in every initial draft as shared scaffold components to maintain network connectivity but were not treated as genome-supported variable reactions.

Predicted growth was evaluated in a vaginal-like synthetic medium with an uptake bound of 1000. A model was considered feasible when its biomass objective value was at least 0.001. For infeasible draft models, rescue candidates were restricted to accessory GPR-associated reactions that had been removed from the corresponding draft. No reactions outside the pan-GEM were considered. Candidate reactions were ranked in descending order of prevalence across the 80 assemblies, with reaction ID used as a deterministic tie-breaker. Reactions were restored sequentially until the biomass objective reached at least 0.001. Backward elimination was then performed in reverse addition order to remove restored reactions that were not required to maintain feasibility. The resulting reaction sets were therefore described as greedily minimized feasibility-rescue sets.

As a sensitivity analysis, we used mixed-integer linear programming (MILP) to identify rescue sets containing the minimum number of reactions within the defined candidate pool. The same biomass objective threshold of 0.001 was used to define model feasibility in both the greedy and MILP analyses. When alternative solutions contained the same minimum number of reactions, reactions with higher prevalence across the 80 genomes were preferred. No reaction stoichiometry, directionality, or GPR rule was changed during ssGEM generation. The complete rescue records and the comparison between the greedy and MILP solutions are provided in Table S4.

No-GPR scaffold reactions and rescued reactions were retained in the final executable ssGEMs and were therefore available during FBA, pFBA, and gene-deletion analyses. However, both categories were excluded from the genome-projected reaction presence/absence matrix and from interpretations of genome-projected reaction variation. Final ssGEMs were checked for biomass feasibility and SBML round-trip consistency. Energy-generating cycles were assessed by testing ATP maintenance under closed-uptake conditions, and MEMOTE v0.17.0 reports were generated for technical quality assessment [29,30,31,32]. ssGEM generation, including the prevalence-ranked greedy feasibility-rescue procedure, was performed using Python v3.9.23, COBRApy v0.29.1, and GLPK v5.0. The MILP rescue sensitivity analysis was performed separately using Python v3.7.12, COBRApy v0.21.0, optlang v1.4.4, and CPLEX v12.10.0.0 (IBM Corporation, Armonk, NY, USA).

### 2.5. Gene Essentiality and Nutrient-Boundary Simulations

A vaginal-like synthetic medium was defined for model feasibility testing, gene essentiality analysis, and nutrient-boundary comparison. The medium was constructed with reference to the synthetic vaginal medium used in the *Gardnerella* pan-GEM study and vaginal metabolomics studies describing nutrient differences across healthy and VVC-associated vaginal environments [11,33,34]. Basic inorganic compounds and nutrients were selected with reference to the published vaginal medium and mapped to exchange reactions shared across the *C. albicans* ssGEM collection. Exchange reactions not included in the vaginal-like synthetic medium were closed to uptake.

Experimental essentiality references were obtained from the *Candida* Genome Database, GRACE, and GRACEv2 [35,36,37]. Genes with clear essential (E) or nonessential (NE) labels were retained. The reference dataset contained 621 essential and 2228 nonessential genes, of which 119 essential and 337 nonessential genes could be mapped to GPR associations in the pan-GEM. Single-gene deletion was performed for the pan-GEM and each ssGEM. A gene was classified as model-essential when its deletion reduced the biomass objective to less than 5% of the value in the corresponding unperturbed model; a 20% cutoff was also evaluated as a sensitivity analysis. Agreement with the experimental labels was assessed using sensitivity, specificity, precision, negative predictive value, accuracy, balanced accuracy, F1 score, and Matthews correlation coefficient. For ranking-based evaluation, a continuous essentiality score was calculated as one minus the ratio of the post-deletion objective value to the corresponding unperturbed objective value and was used to calculate the area under the receiver operating characteristic curve and average precision. Because the experimental data and analyzed assemblies did not necessarily represent the same genetic backgrounds, the results were interpreted as consistency assessments rather than strain-matched validation. The complete gene essentiality results and performance statistics are provided in Table S5.

D-glucose and L-glutamate were selected for the nutrient-boundary simulations by cross-referencing metabolites reported in vaginal metabolomics studies with the exchange reactions available in the final models [33,34]. Seven conditions were evaluated. In the standard baseline condition, the uptake bounds for D-glucose and L-glutamate were both set to 1. The uptake bound for D-glucose, L-glutamate, or both nutrients was then increased to 10. A restricted reference condition was defined by setting both uptake bounds to 0.1. Two additional conditions were evaluated by setting D-glucose to 10 and L-glutamate to 0.1, or D-glucose to 0.1 and L-glutamate to 10. The uptake bounds for all other medium components remained at 1 in these seven conditions. The complete medium composition and nutrient-condition definitions are provided in Table S6. Only models with feasible objectives under both conditions of a comparison were included in paired summaries. FBA was performed under all seven nutrient conditions. pFBA, with the FBA optimum fixed at 100%, was additionally performed under the four main conditions: baseline, increased D-glucose uptake, increased L-glutamate uptake, and increased uptake of both nutrients. The exchange-flux comparison used the baseline and both-increased conditions.

### 2.6. Statistical Analysis

Orthogroup-content variation was summarized using principal component analysis (PCA). Pairwise orthogroup-content and genome-projected reaction-content differences were quantified using Jaccard distance. The association between the two distance matrices was evaluated using two-sided Mantel permutation tests with Pearson and Spearman correlation statistics. Genome labels in one distance matrix were randomly permuted 9999 times, and permutation *p* values were calculated as (*b* + 1)/(9999 + 1), where *b* was the number of permuted statistics with an absolute value at least as large as the observed statistic. A fixed random seed of 2026 was used.

Paired comparisons of predicted growth between nutrient conditions were performed using two-sided Wilcoxon signed-rank tests. *p* values were adjusted using the Holm method across five prespecified contrasts among the four main conditions: increased D-glucose uptake versus baseline, increased L-glutamate uptake versus baseline, increased uptake of both nutrients versus baseline, increased uptake of both nutrients versus increased D-glucose uptake, and increased uptake of both nutrients versus increased L-glutamate uptake. Only ssGEMs that were feasible under both conditions of a comparison were included. Predicted growth differences with an absolute value no greater than 10^−8^ were classified as unchanged.

For visualization of pFBA flux differences between the baseline and both-increased conditions, the 15 exchange reactions with the largest median absolute differences across the ssGEMs were selected. Flux differences were calculated as both increased minus baseline, transformed using the inverse hyperbolic sine, and hierarchically clustered using Euclidean distance and average linkage. The pFBA calculations were performed using Python v3.7.12, COBRApy v0.21.0, optlang v1.4.4, and CPLEX v12.10.0.0. Subsequent statistical analyses and visualization were performed using Python v3.9.23, pandas v2.3.1, NumPy v2.0.2, SciPy v1.13.1, scikit-learn v1.6.1, Matplotlib v3.9.1, and seaborn v0.13.2.

## 3. Results

### 3.1. Pan-Genome Analysis of 80 C. albicans Genomes

**Figure 2:**
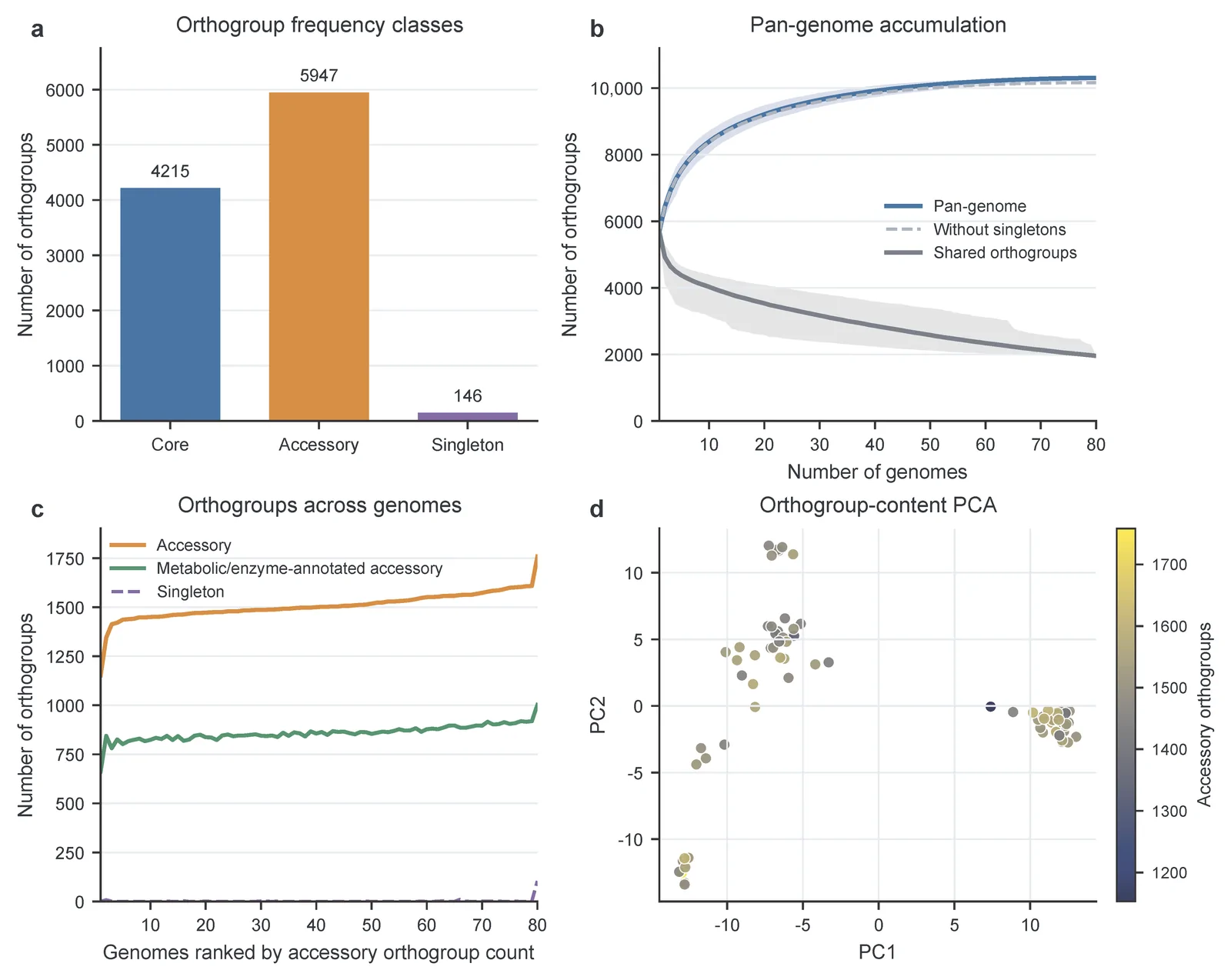
Pan-genome structure of 80 public *C. albicans* genomes. (**a**) Numbers of core, accessory, and singleton orthogroups. (**b**) Pan-genome and shared-orthogroup accumulation curves based on 999 random assembly addition orders. Lines represent mean values, and shaded areas indicate 95% empirical intervals. (**c**) Numbers of accessory, metabolic/enzyme-annotated accessory, and singleton orthogroups in each genome, ordered by total accessory orthogroup count. (**d**) PCA of orthogroup presence/absence profiles showing gene-content variation across the 80 genomes.

After quality control, 80 public *C. albicans* genome assemblies entered pan-genome analysis (Table S1). OrthoFinder identified 10,308 orthogroups in total, of which 4215 were core, 5947 accessory, and 146 singletons (Figure 2a). Among the core orthogroups, 1954 were present in all 80 genomes.

The pan-genome accumulation curve continued to increase but gradually flattened as more assemblies were added (Figure 2b). Across the final ten assembly addition steps, the mean number of newly added orthogroups decreased from 5.06 at assembly 71 to 1.98 at assembly 80. When singletons were excluded, the corresponding value decreased from 2.86 to 0. Meanwhile, the number of orthogroups shared by all included assemblies declined to 1954. These patterns indicate slow continued expansion and an approach to a plateau within the analyzed assembly collection.

Individual genomes contained 4657–6024 orthogroups (median 5703). Each genome carried 3305–4214 core orthogroups (median 4203), 1153–1758 accessory orthogroups (median 1501), and 0–104 singleton orthogroups (median 0) (Figure 2c). Most genomes carried few or no singleton orthogroups, whereas singleton counts were concentrated in a small number of samples.

PCA of the orthogroup presence/absence matrix revealed structured dispersion of the 80 *C. albicans* genomes along PC1 and PC2 in gene-content space, rather than a single overlapping cluster (Figure 2d). PC1 separated the major axis of gene-content variation, and genomes with negative PC1 values further spread along PC2. Accessory burden was broadly comparable across these regions, indicating that this structure arises from combinatorial differences across multiple accessory orthogroups.

### 3.2. Functional Annotation and Distribution of Candida-Associated Orthogroups Across the Pan-Genome

Among the 10,308 orthogroups, 9024 contained at least one broad functional annotation in the aggregated eggNOG-mapper results. Enzyme or metabolic annotations were identified for 2812 core, 3360 accessory, and 90 singleton orthogroups. KEGG reaction annotations were identified for 786 core, 729 accessory, and 19 singleton orthogroups. These results indicate that metabolically interpretable gene families are not confined to the core genome but are also broadly represented within the accessory pool.

**Figure 3:**
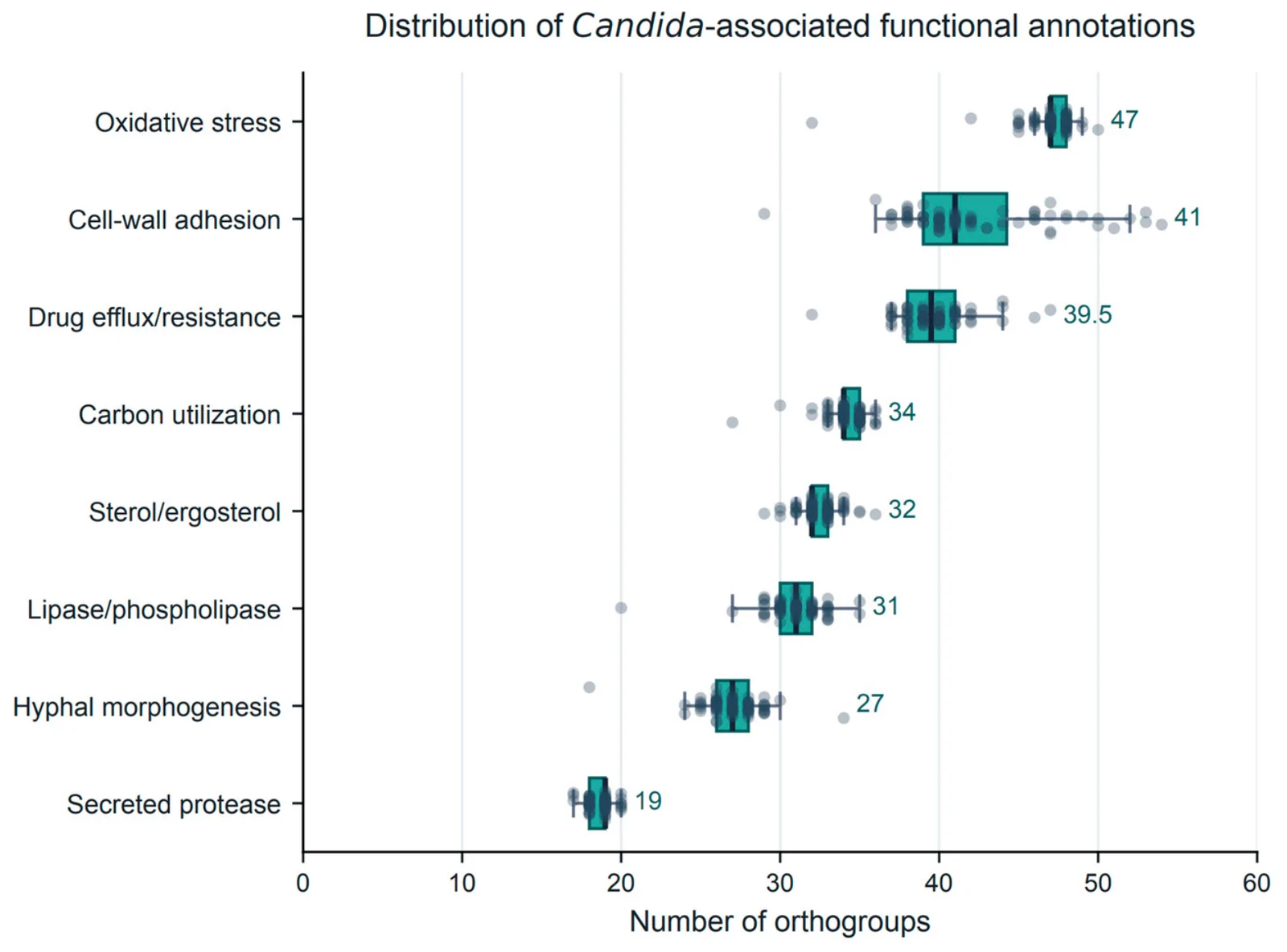
Distribution of *Candida*-associated functional annotations across 80 *C. albicans* genomes. Boxplots show the numbers of orthogroups assigned to eight functional categories in each genome. Points represent individual genomes, and numerical labels indicate category medians.

To further characterize functional cues relevant to *Candida* biology across the pan-genome, we performed a *Candida*-associated functional feature screen on the orthogroups. In total, 522 unique orthogroups were assigned to at least one of eight functional categories, including cell-wall adhesion, hyphal morphogenesis, drug efflux/resistance, oxidative stress, lipase/phospholipase, carbon utilization, sterol/ergosterol, and secreted protease. These comprised 202 core, 312 accessory, and eight singleton orthogroups. All eight categories were represented in each of the 80 genomes, although the number of orthogroups assigned to each category varied among genomes (Figure 3). The oxidative-stress category had the highest median count, at 47 orthogroups per genome. Other median counts were 41 for cell-wall adhesion, 39.5 for drug efflux/resistance, 34 for carbon utilization, 32 for sterol/ergosterol metabolism, 31 for lipase/phospholipase activity, 27 for hyphal morphogenesis, and 19 for secreted proteases.

Keyword-based screening for antifungal-relevant candidates identified 110 orthogroups, predominantly drug efflux transporters, with azole target/sterol pathway and echinocandin/cell-wall target genes also detected. Overall, accessory gene variation among *C. albicans* strains is reflected not only in metabolic annotation but also in cell-wall adhesion, stress response, drug efflux, morphological transition, and nutrient utilization—functional categories broadly relevant to *Candida* adaptation. It should be emphasized that these results describe the distribution of functional annotations across the genome collection and do not represent the drug susceptibility or virulence phenotype of any particular strain.

### 3.3. Construction of the C. albicans pan-GEM

**Figure 4:**
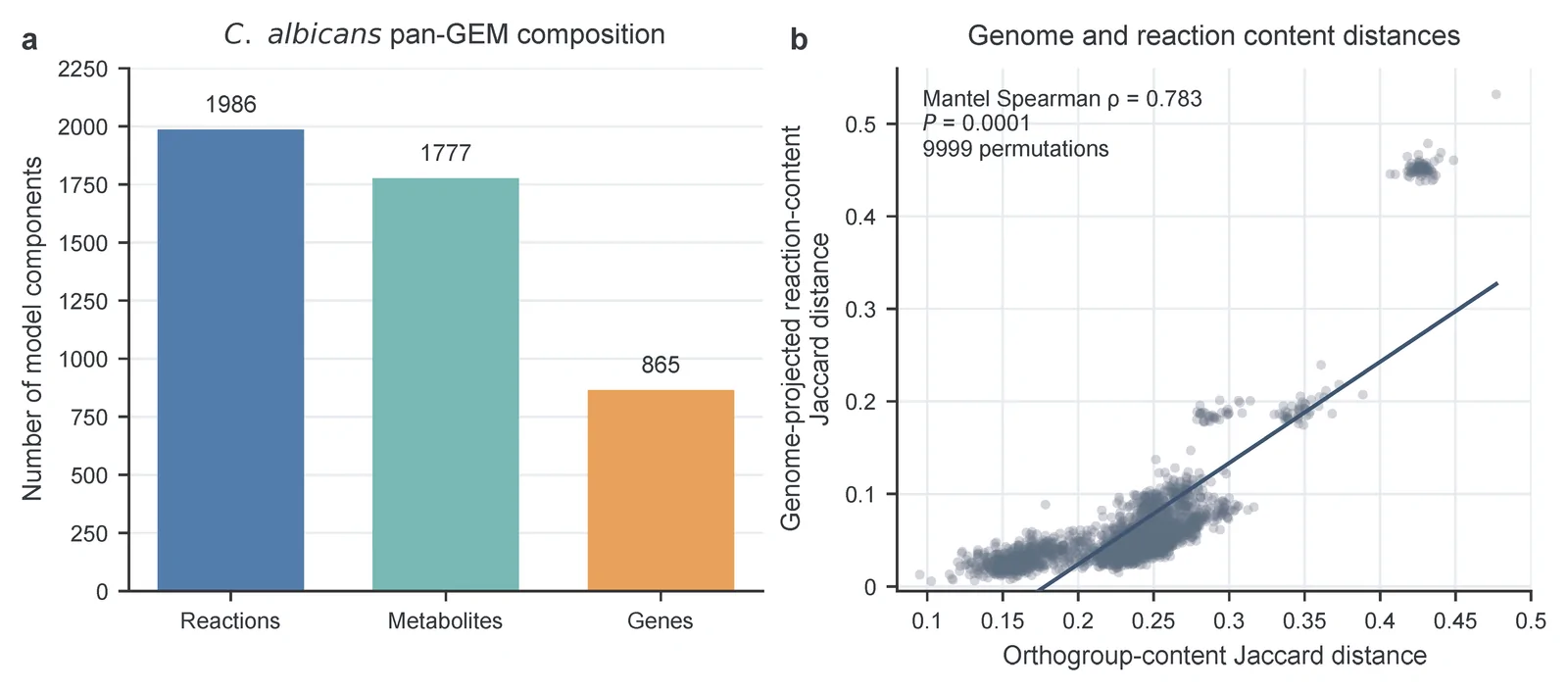
Composition of the *C. albicans* pan-GEM and projection of genome content into reaction space. (**a**) Composition of the final executable *C. albicans* pan-GEM, including reactions, metabolites, and genes. (**b**) Pairwise comparison of orthogroup-content and genome-projected reaction-content Jaccard distances across the 80 genomes. Points represent genome pairs and are shown for visualization. The association between the two distance matrices was evaluated using a two-sided Mantel test with 9999 permutations. The fitted line is shown as a visual guide; statistical significance was assessed using the two-sided Mantel test.

Based on published *C. albicans* GEMs, other fungal models, public reaction resources, and orthogroup-linked GPR evidence, we constructed a *C. albicans* pan-GEM. The model comprises 1986 reactions, 1777 metabolites, and 865 genes (Figure 4a; Table S3). Among the reactions, 1623 have GPR associations and are available for subsequent genome projection, whereas 363 no-GPR scaffold reactions were retained as part of the shared network to maintain connectivity and executability. The final pan-GEM met the predicted growth feasibility criterion under the defined medium and passed the SBML round-trip, closed-uptake energy-generating-cycle, reaction-directionality, and structural-consistency checks. The MEMOTE total score was 0.698. The complete pan-GEM MEMOTE report is available on GitHub (https://github.com/sylviameng0703/C_albicans_pan-GEM, accessed on 13 September 2026)).

Among the 1623 GPR-associated reactions, 512 had GPR support in all 80 genomes, whereas 1111 showed variable GPR support across the genome collection. No-GPR scaffold reactions were excluded from the interpretation of genome-projected reaction variation. The final pan-GEM thus retains both a reaction set that can be projected from genomic evidence and a set of scaffold reactions that maintain the shared network structure.

We compared the orthogroup-content and genome-projected reaction-content Jaccard distance matrices using a Mantel test with 9999 permutations. The two distance matrices were positively associated (Mantel Pearson *r* = 0.791 and Mantel Spearman *ρ* = 0.783; both two-sided *p* = 0.0001; Figure 4b). This result indicates that differences in orthogroup content were associated with differences in genome-projected reaction content. Reaction-content distances were generally lower than orthogroup-content distances because only orthogroups with resolvable GPR mappings contributed to the reaction matrix, and multiple genes or orthogroups could support the same reaction.

### 3.4. Construction of 80 C. albicans ssGEMs

**Figure 5:**
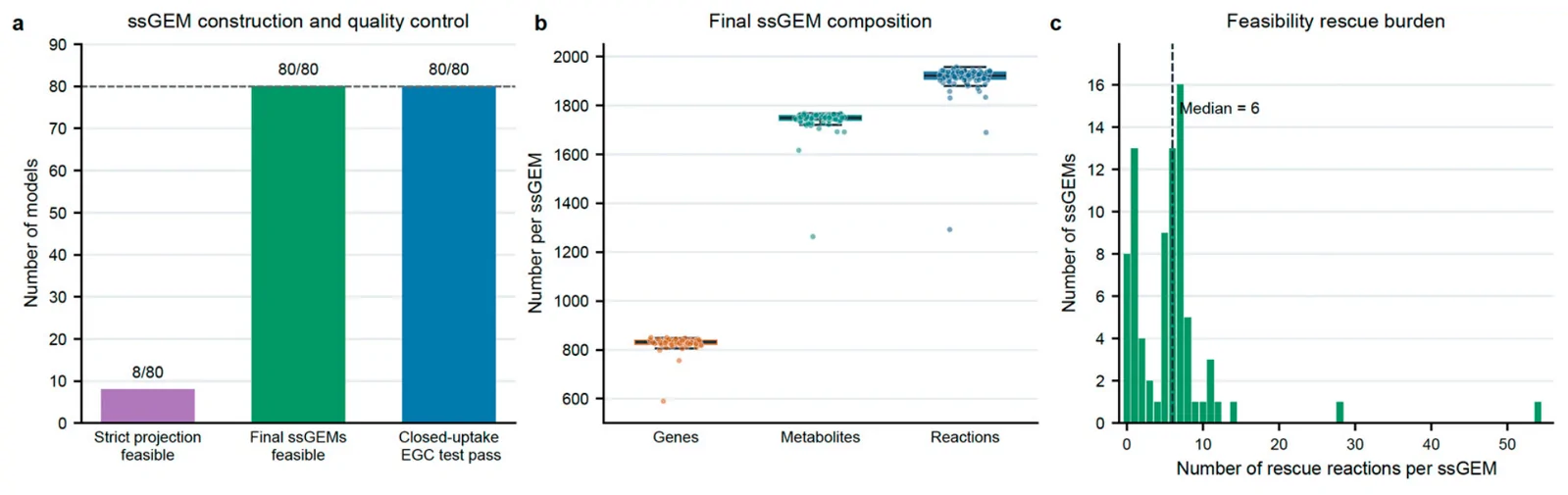
Construction and quality control of the 80 strain-specific GEMs. (**a**) Predicted growth feasibility and closed-uptake energy-generating-cycle test during ssGEM generation, including strict genome-projected drafts and final models after greedily minimized feasibility rescue. (**b**) Distribution of reaction, metabolite, and gene counts across the final 80 ssGEMs. Each point represents one strain-specific model. (**c**) Distribution of the numbers of feasibility-rescue reactions across the ssGEMs. Rescue reactions were tracked separately from genome-projected reaction evidence.

Using the final pan-GEM as the template, we generated draft ssGEMs by retaining reactions supported by each genome’s orthogroup-linked GPR evidence, together with the shared no-GPR scaffold reactions. Eight of the 80 strict genome-projected drafts met the predicted growth feasibility criterion. The remaining 72 drafts required greedily minimized feasibility rescue using accessory GPR-associated reactions that had been removed during genome projection. All 80 final ssGEMs met the predicted growth feasibility criterion and passed the closed-uptake energy-generating-cycle test (Figure 5a).

The final ssGEMs contained 1292–1957 reactions (median, 1922), 1263–1767 metabolites (median, 1748.5), and 590–850 genes (median, 831) (Figure 5b). Individual models required 0–54 rescued reactions, with a median of six (Figure 5c). Across the ssGEM collection, the 466 rescue events all involved accessory GPR-associated reactions from the pan-GEM. No reactions from outside the pan-GEM were introduced. Rescued reactions were recorded separately and were excluded from the interpretation of genome-projected reaction variation.

Removing all 363 no-GPR scaffold reactions simultaneously rendered all 80 ssGEMs unable to meet the biomass-feasibility criterion, indicating that this shared reaction set collectively supported biomass production or network connectivity. Inclusion of rescued reactions reduced the median pairwise reaction-content Jaccard distance from 0.0471 under strict genome projection to 0.0437, corresponding to a reduction of 7.2%. Pairwise distances before and after rescue remained strongly correlated (Spearman’s *ρ* = 0.9964).

The MILP sensitivity analysis identified 463 rescue events. Among the 72 models requiring rescue, 71 had the same rescue-set size under the greedy and MILP methods. For GCA_014825715, the number decreased from 54 to 51. Seventeen models had different rescue reaction sets, although 16 of them contained the same number of reactions under both methods. All models obtained using the MILP rescue sets also supported predicted growth and passed the closed-uptake energy-generating-cycle test.

All 80 final ssGEMs completed MEMOTE assessment. The mean total score was 0.695, with a median of 0.696 and a range of 0.688–0.702 (Table S4). The complete MEMOTE reports are available on GitHub (https://github.com/sylviameng0703/C_albicans_pan-GEM, accessed on 13 September 2026)).

### 3.5. Gene Essentiality Predictions in a Vaginal-like Synthetic Medium

The GRACE and GRACEv2 reference dataset contained 621 experimentally essential and 2228 experimentally nonessential *C. albicans* genes [36,37]. Among these genes, 119 essential and 337 nonessential genes had resolvable GPR associations in the pan-GEM. Using a maximum uptake bound of 1000 and a 5% threshold relative to the unperturbed objective value, the pan-GEM predicted 23 of the 119 experimentally essential genes as essential and 325 of the 337 experimentally nonessential genes as nonessential. This corresponded to a sensitivity of 0.193, specificity of 0.964, precision of 0.657, accuracy of 0.763, balanced accuracy of 0.579, and Matthews correlation coefficient of 0.260. The AUROC was 0.603, and the average precision was 0.377 (Table S5). Although sensitivity and overall discrimination were limited, the high specificity and moderate precision indicate that the pan-GEM recovered a conservative subset of experimentally supported essential genes represented in the metabolic network.

Under the same conditions, all 80 ssGEMs maintained feasible baseline growth. Thirty-two experimentally supported genes were predicted as essential in at least one ssGEM, of which 23 were model-essential across all 80 ssGEMs (Figure S1; Table S5). These universal model-essential genes included genes involved in several conserved metabolic modules: ergosterol biosynthesis genes *ERG11*, *ERG12*, *ERG13*, *ERG27*, *HMG1*, *MVD*, and *IDI1*; fatty acid biosynthesis genes *FAS1* and *FAS2*; amino-sugar/cell-wall precursor biosynthesis genes *GFA1* and *GNA1*; and branched-chain amino acid, aromatic amino acid, and tryptophan biosynthesis genes *ILV2*, *ILV5*, *ARO1*, *ARO2*, and *TRP2-TRP5*. The no-GPR scaffold reactions lacked gene associations and were therefore not directly evaluated by gene deletion, but they remained part of the shared network context used for the analysis. Accordingly, the 23 universal model-essential calls describe dependencies within the final executable ssGEMs rather than within the strictly genome-projected reaction layer alone.

Increasing the essentiality cutoff to 20% yielded the same 23 genes across all 80 ssGEMs. Reducing the uptake bound to one yielded the same 23 genes across all 79 feasible ssGEMs (Table S5). Because the experimental screens and the analyzed genomes were not strain-matched and were evaluated under different environmental conditions, these results should be interpreted as a consistency assessment rather than strain-specific experimental validation.

### 3.6. Predicted Growth Responses to D-Glucose and L-Glutamate Uptake Constraints

Using the vaginal-like synthetic medium, we evaluated seven combinations of D-glucose and L-glutamate uptake bounds while keeping all other medium constraints unchanged. The standard baseline uptake bound was one for both nutrients. The main comparisons separately increased D-glucose, L-glutamate, or both uptake bounds to 10. A restricted reference condition and two additional release conditions were also evaluated. Candidate nutrients were selected based on directional evidence from VVC/RVVC-related vaginal metabolomics studies, but only metabolites that could be mapped unambiguously to exchange reactions in all final models were considered [33,34] (Table S6).

Of the 80 ssGEMs, 79 were feasible under all four main conditions and were included in the paired comparisons. GCA_014825715 was infeasible under these conditions and was excluded. At baseline, the median predicted growth was 0.604. Increasing the D-glucose uptake bound increased the median to 1.270; all 79 ssGEMs showed an increase, with a median difference of 0.666 and a median fold change of 2.10 (two-sided Wilcoxon signed-rank test, Holm-adjusted *p* = 5.74 × 10^−14^). Increasing the L-glutamate uptake bound produced a smaller change, with the median increasing from 0.604 to 0.611. Predicted growth increased in 69 ssGEMs and remained unchanged in 10, with a median difference of 0.0058 and a median fold change of 1.010 (Holm-adjusted *p* = 8.35 × 10^−14^). When both uptake bounds were increased, all 79 ssGEMs showed higher predicted growth than at baseline, with a median of 1.270, a median difference of 0.666, and a median fold change of 2.10 (Holm-adjusted *p* = 5.74 × 10^−14^). Compared with increasing D-glucose alone, increasing both nutrients produced no median change, although 30 ssGEMs showed small additional increases. These results indicate that the predicted growth response was mainly associated with the D-glucose uptake constraint, whereas increasing L-glutamate had a smaller additional effect.

**Figure 6:**
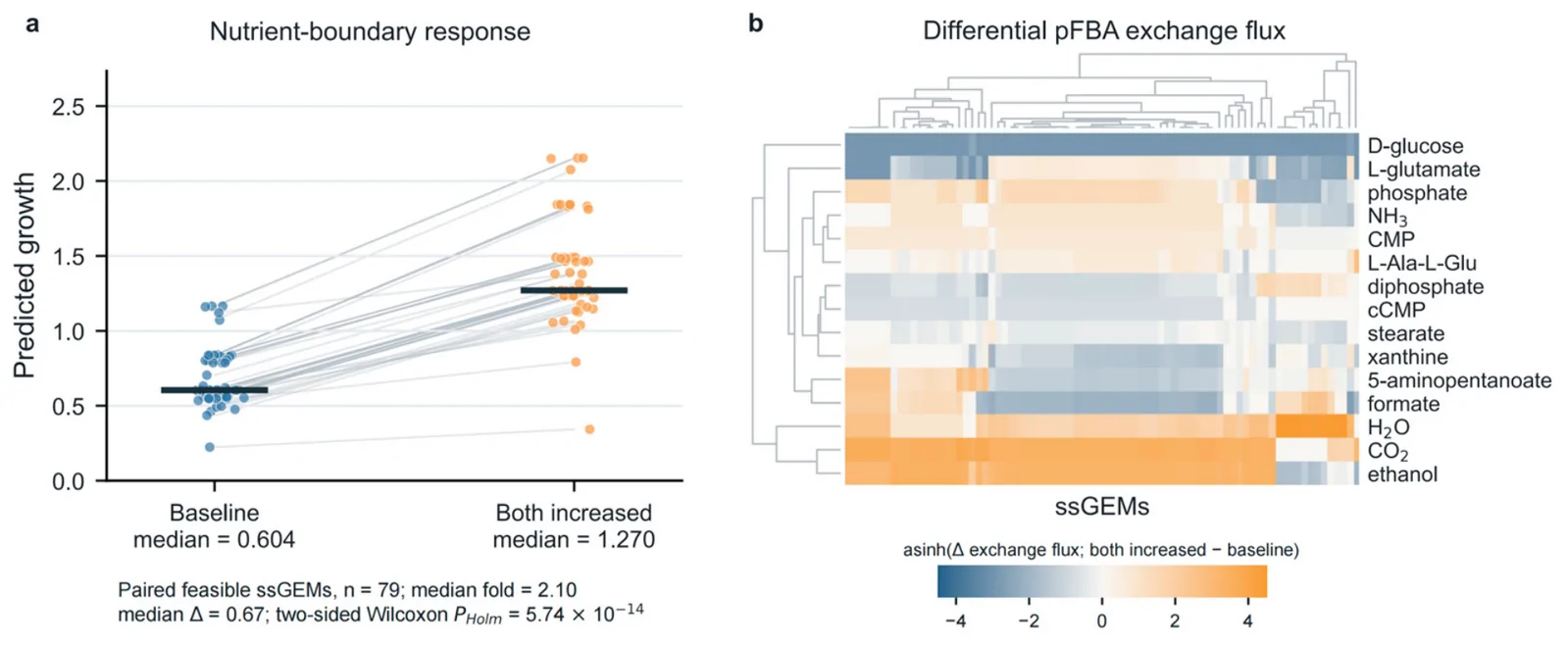
Predicted responses of the ssGEMs to increased D-glucose and L-glutamate uptake bounds. (**a**) Paired predicted growth of 79 feasible ssGEMs under the baseline condition, in which the uptake bounds for D-glucose and L-glutamate were both set to 1, and the both-increased condition, in which both bounds were set to 10. Lines connect predictions from the same ssGEM, and horizontal bars indicate medians. The *p* value was calculated using a two-sided Wilcoxon signed-rank test with Holm correction. (**b**) Hierarchically clustered pFBA flux differences for the 15 exchange reactions with the largest median absolute changes between the two conditions. Columns represent ssGEMs. Flux differences were calculated as both increased minus baseline and transformed using the inverse hyperbolic sine for visualization. Negative differences indicate a shift toward greater uptake or lower secretion, whereas positive differences indicate a shift toward greater secretion or lower uptake.

The pFBA comparison between the baseline and both-increased conditions identified changes in several exchange fluxes (Figure 6b; Table S6). All 79 ssGEMs showed a shift toward greater D-glucose uptake, consistent with the imposed increase in its uptake bound. Exchange fluxes involving CO2, ethanol, H2O, L-glutamate, and other metabolites varied in magnitude and, for some reactions, direction across the ssGEMs. Figure 6b presents the 15 exchange reactions with the largest median absolute flux differences. These results characterize the predicted exchange-flux responses of the ssGEMs under the defined nutrient constraints.

## 4. Discussion

*C. albicans* shows substantial intraspecific genomic diversity, but the metabolic consequences of this diversity have not been systematically organized in a comparable modeling framework. In this study, we reconstructed a species-level *C. albicans* pan-GEM and generated 80 ssGEMs through genome projection, with feasibility rescue required for 72 of the initial drafts. The resulting ssGEM collection shows that most model-supported metabolic functions are conserved across the analyzed genomes, while a smaller set of GPR-associated reactions varies with accessory gene content. This structure provides a practical framework for separating conserved metabolic functions from genome-projected reaction differences.

The pan-genome results showed a conserved core gene pool together with a large accessory component. This pattern is consistent with fungal pan-genome studies showing that non-core genes may contribute to environmental adaptation and host-associated functions [13]. Comparative genomic and transcriptomic analyses have also linked natural variation among *C. albicans* strains to cell-wall proteins, morphogenesis, antifungal responses, and other pathogenicity-related traits [38,39]. In our dataset, accessory orthogroups included enzyme or metabolic annotations and annotations associated with cell-wall adhesion, drug efflux/resistance, oxidative stress, carbon utilization, and morphogenesis. These results indicate that the accessory genome contains gene families with potentially relevant functional annotations, although these annotations do not establish drug susceptibility or virulence phenotypes.

The pan-GEM construction converted this gene-content variation into a curated reaction framework. The refined *C. albicans* GEM reported by Hu et al. was used as the primary model scaffold [10]. Published *C. albicans* GEMs, other fungal models, and public reaction databases were used to review reaction identities, metabolite identities, GPRs, compartmentalization, and directionality [8,9,22,23,24,25,26,27]. This evidence-based curation is important because compartmentalized metabolism, lipid biosynthesis, cofactor balance, and transport reactions strongly affect the feasibility of fungal metabolic models. The final pan-GEM therefore represents a curated reaction framework rather than a simple union of database reactions.

A Mantel test showed a positive association between orthogroup-content distance and genome-projected reaction-content distance (Spearman’s *ρ* = 0.783, *p* = 0.0001; 9999 permutations), indicating that part of the orthogroup variation was retained after GPR projection. This finding is consistent with previous pan-genome-scale metabolic modeling studies, in which genomic variation was translated into comparable reaction-level differences [11,12,14]. However, this association represents a relationship within the genome-to-model projection rather than independent phenotypic validation, because reaction content was derived from orthogroup presence/absence through GPR rules. The smaller range of reaction-content distances suggests that many gene-family differences were not represented in the executable reaction network [40]. This compression may reflect isoenzymes, alternative GPR structures, and accessory genes without resolved metabolic reaction mappings.

The final ssGEM collection represented both reaction-level variation and conserved model-predicted metabolic dependencies. Reaction counts differed among models, and variable GPR-associated reactions covered redox and cofactor, lipid and sterol, amino acid, central-carbon, and cell-wall and glycan metabolism. Twenty-three genes classified as experimentally essential in the reference dataset were predicted to be essential in all 80 ssGEMs. These genes were involved in ergosterol, fatty acid, amino-sugar, branched-chain amino acid, aromatic amino acid, and tryptophan biosynthesis. Experimental studies further support the biological importance of several of these pathways. For example, deletion of *ILV2* causes severe amino acid starvation phenotypes and reduces virulence in *C. albicans*, whereas loss of *GNA1* reduces fungal survival and virulence in vivo [41,42]. Genome-wide essentiality screens in *C. albicans* provide relevant experimental context for these model predictions [36,37]. The conserved predictions also included *ERG11*, the target of azole antifungal drugs [43]. These results indicate that the ssGEM collection recovers several previously reported metabolism-related dependencies and identifies candidates for further experimental evaluation [8].

The vaginal-like synthetic medium simulations provide an application example for the ssGEM collection. Based on vaginal metabolomics studies, we varied only D-glucose and L-glutamate uptake bounds [33,34]. Increasing the D-glucose uptake bound produced a clear increase in predicted growth across the 79 feasible ssGEMs, whereas increasing L-glutamate produced a much smaller change. Increasing both uptake bounds resulted in a median response similar to that observed when D-glucose alone was increased. The pFBA analysis further showed consistent changes in D-glucose exchange flux and more variable changes in other exchange fluxes across the ssGEMs. These results indicate that D-glucose uptake was the main nutrient constraint under the tested conditions and demonstrate the use of the ssGEM collection for comparative nutrient-response analysis.

This study also has limitations. Orthogroup presence was used as an approximation for gene presence, and we did not perform protein-level completeness screening for every GPR-associated gene. In addition, the public genome assemblies were accompanied by incomplete and uneven metadata, which limited robust subgroup analyses according to isolation source, geographic origin, or clinical status. Although the pan-GEM integrates multiple *C. albicans* and fungal models, parts of fungal lipid metabolism, glycosylation, cofactor metabolism and compartmentalized transport may remain incomplete. The experimental essentiality datasets were generated using strains and conditions that did not necessarily correspond to the genomes and vaginal-like synthetic medium used here, and only genes with resolvable GPR associations could be evaluated. The vaginal-like synthetic medium was designed for model comparison rather than for reconstructing vaginal fluid concentrations, pH, redox state or host immune activity. Furthermore, pFBA provides one parsimonious solution, and alternative optimal solutions may produce different flux distributions. Future work combining quantitative metabolomics, transcriptomics, strain growth assays, gene-knockout data and co-culture experiments will be needed to test the predicted exchange-flux responses, conserved predicted essential genes and nutrient-response differences.

Together, this work establishes a curated *C. albicans* pan-GEM and a collection of 80 ssGEMs that connect public genome variation to genome-projected reaction differences. The framework highlights a broadly conserved metabolic core, dentifies model-predicted essential metabolic functions for further experimental evaluation, and provides a reusable basis for future condition-specific modeling of *C. albicans* metabolism.

## 5. Conclusions

This study constructed a curated *C. albicans* pan-GEM and 80 ssGEMs by projecting public genome assemblies with feasibility rescue. By linking orthogroup variation, GPR-based reaction projection and feasibility testing, this work provides a traceable basis for comparing conserved metabolic functions and genome-projected reaction differences. The final models highlight a conserved metabolic core, accessory reaction variation across multiple functional modules, and a set of metabolic genes consistently predicted as essential across the ssGEM collection. These resources provide a foundation for future comparative modeling of *C. albicans* metabolism, condition-specific vaginal microbiome interactions, and experimental evaluation of metabolic dependencies predicted in silico.

## Data Availability

The final *C. albicans* pan-GEM, 80 strain-specific GEMs, MEMOTE reports, source data tables and scripts used to generate the ssGEMs are available on GitHub at https://github.com/sylviameng0703/C_albicans_pan-GEM (accessed on 13 September 2026). Public genome accession information is provided in Table S1.

## Supplementary Materials

The following supporting information can be downloaded at https://www.mdpi.com/article/10.3390/jof12090697/s1. Figure S1: Experimental-reference genes with model-essential calls across the ssGEM collection; Table S1: Metadata and genome quality-control information for the 80 public *C. albicans* genomes; Table S2: Orthogroup classifications, functional-screening criteria, and matched orthogroups; Table S3: Final *C. albicans* pan-GEM reaction, metabolite, and gene information; Table S4: Greedy and MILP feasibility-rescue records, rescue comparison, and ssGEM MEMOTE scores; Table S5: Gene essentiality results and performance statistics; Table S6: Vaginal-like synthetic medium composition, nutrient-condition definitions, predicted growth comparisons, and pFBA exchange-flux results.
