# Supplementary material for: Pan-Genome-Scale Metabolic Reconstruction Reveals Conserved Metabolic Functions in Candida albicans
Source: J Fungi (Basel). 2026 Sep 17;12(9):697. doi: 10.3390/jof12090697 (PMC13608804; doi:10.3390/jof12090697)
Supplement: Supplementary file 1 [file jof-12-00697-s001.zip › supplementary figure.pdf]

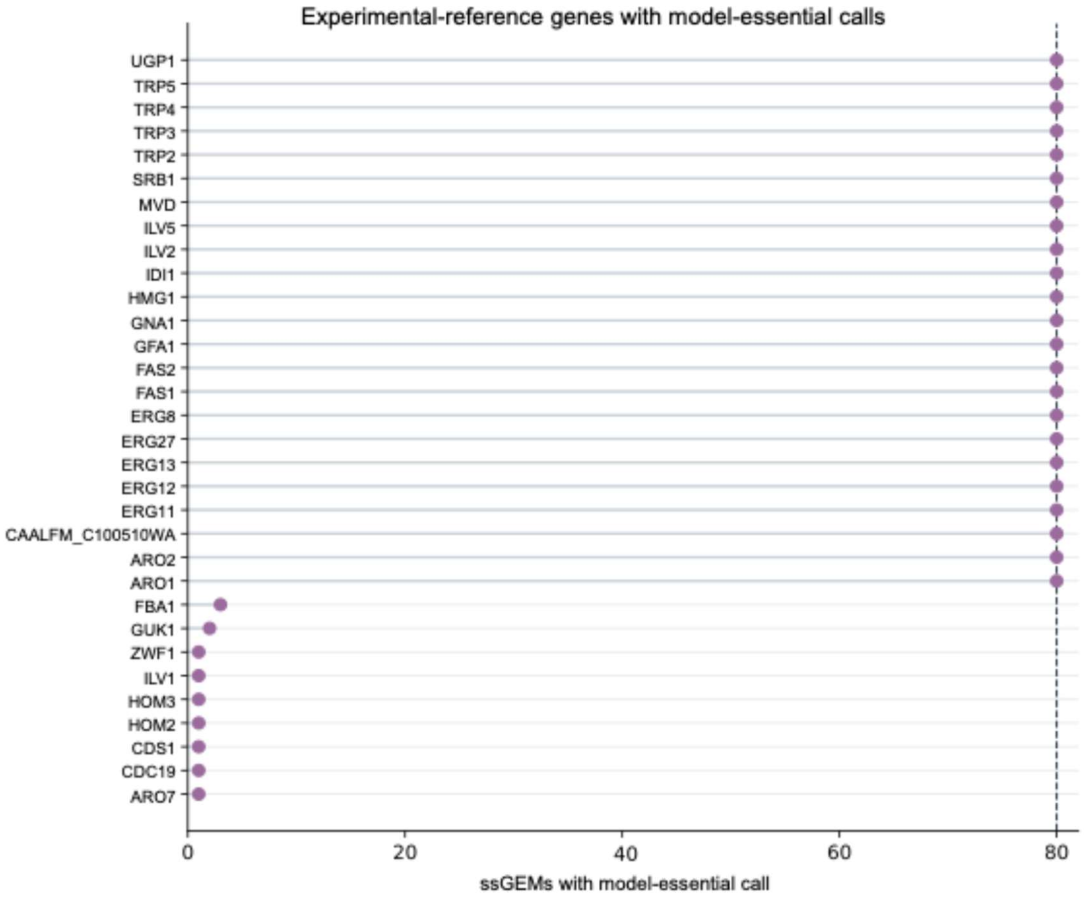

Figure S1. Experimentally essential reference genes predicted as model-essential in at least one ssGEM. Points indicate the number of final ssGEMs in which each gene was predicted as essential under the main essentiality condition, with an uptake bound of 1000 and a 5% essentiality cutoff. The dashed line marks all 80 ssGEMs.
